# Psychological capital matters: a methodological proof-of-concept for dual-dimensional student competitiveness assessment

**DOI:** 10.3389/fpsyg.2026.1811331

**Published:** 2026-08-17

**Authors:** Qicui Du, Long Xu

**Affiliations:** 1 School of Stomatology, Qilu Medical University, Zibo, Shandong, China; 2 School of Basic Medical Sciences, Qilu Medical University, Zibo, Shandong, China

**Keywords:** behavioral competencies, career construction theory, fuzzy analytic hierarchy process, higher education policy, holistic assessment, psychological capital, signaling theory, sustainable employability

## Abstract

**Introduction:**

In the contemporary era of boundaryless careers and Industry 5.0, sustainable employability refers not only to obtaining an initial job but also to maintaining and developing meaningful employment through adaptable human, social, and psychological resources. Traditional evaluation systems in higher education continue to rely heavily on visible cognitive signals such as cumulative grade point average (CGPA), thereby creating a hard-skill bias and leaving psychosocial resources weakly signaled. This study proposes a Balanced Weight-Constrained Fuzzy Analytic Hierarchy Process (BW-FAHP) as a methodological proof-of-concept for dual-dimensional student competitiveness assessment.

**Methods:**

The publicly available Kaggle campus-placement dataset (*N* = 215) was used as an empirical seed for academic indicators, and a bootstrap-based simulation dataset (*N* = 10,000) was generated to examine the internal behavior of the scoring model. Several psychosocial indicators and the placement criterion were proxy-based or simulated rather than directly observed.

**Results:**

Imposing a transparent equilibrium between cognitive-academic and psychosocial-behavioral indicators changed candidate rankings relative to a GPA-centric benchmark and revealed a potentially overlooked high-potential profile characterized by average academic scores but strong behavioral resources. The balanced score also showed stronger alignment with the simulated placement criterion than the GPA-centric benchmark.

**Discussion:**

The stronger alignment should be interpreted as internal criterion alignment under the stated simulation assumptions rather than as evidence of real-world predictive superiority. The study provides a transparent and reproducible framework for making soft-skill assumptions explicit and offers a foundation for future validation using primary longitudinal data, validated psychosocial measures, and formal expert elicitation.

## 1 Introduction

The global labor market is currently navigating a period of unprecedented disruption and transformation, a phenomenon frequently characterized by scholars and policymakers as the “Fourth Industrial Revolution” (Industry 4.0) transitioning rapidly into “Society 5.0” (Yang and Omar, 2026). In this volatile, uncertain, complex, and ambiguous (VUCA) landscape, the traditional paradigms of professional success, career longevity, and workforce readiness are being fundamentally rewritten. Historically, the dominant “learn-to-work” model presumed that a static set of technical skills and explicit knowledge acquired during formal university education—often encapsulated in a specific degree—would suffice to sustain a professional career spanning three to four decades. However, the accelerating pace of technological innovation, particularly the ubiquity of automation, artificial intelligence, and machine learning, has rendered this linear model obsolete. Recent empirical studies, such as those by Wen et al. (2025), indicate that the “half-life” of a learned technical skill has shrunk to less than 5 years, creating a perpetual cycle of obsolescence. Consequently, the construct of “employability” has shifted from a static possession of qualifications to a dynamic capacity for “sustainable employability”—defined not merely as the ability to secure entry-level employment but as the continuous capacity to adapt, learn, unlearn, and maintain psychological wellbeing across a turbulent and boundaryless career lifespan (Subas et al., 2022; Salazar et al., 2025).

Despite the growing consensus among organizational psychologists and human resource practitioners regarding the critical market demand for adaptive, resilient, and psychologically robust employees, higher education institutions (HEIs) globally face a persistent and deepening “criterion problem.” Universities are traditionally structured around the transmission, verification, and certification of explicit, cognitive knowledge, broadly categorized as “Human Capital” (Becker, 1964). The entire infrastructure of higher education evaluation relies heavily on standardized testing metrics, Cumulative Grade Point Average (CGPA), and the accumulation of technical certifications. These metrics are favored not necessarily for their predictive validity regarding long-term career success, but because they are objective, transparent, quantifiable, and easily rankable (Nagy, 2025). This institutional reliance creates a pervasive phenomenon known as “hard-skill bias” or “measurability bias” (Gottwald et al., 2024). This bias unintentionally but systematically marginalizes the “soft,” intangible, yet vital competencies such as emotional intelligence, resilience, communicative agility, and collaborative synergy—attributes that constitute the core of “Psychological Capital” (PsyCap).

The consequences of this misalignment are profound and multifaceted. As highlighted by Rao (2013) and D’Auria and Stanton (2023), the labor market is currently witnessing a paradoxical “skills gap”: a significant proportion of university graduates possess impeccable academic credentials and high technical proficiency yet fail to integrate successfully into complex organizational environments. Employers frequently report that while high-GPA graduates demonstrate superior cognitive processing abilities, they often lack the psychosocial robustness to cope with failure, navigate ambiguity, or collaborate effectively in diverse, cross-functional teams (Shayrine and Venugopal, 2024). This discrepancy suggests that the current signaling mechanisms used in graduate recruitment are malfunctioning.

From the theoretical lens of Signaling Theory (Spence, 1973), education serves as a signal of unobservable productivity to prospective employers. In a functioning market, a high GPA serves as a costly, hard-to-fake signal of a candidate’s discipline and cognitive ability. However, in the current VUCA context, this signal has become increasingly “noisy” and insufficient. The “measurability bias” in university assessments means that GPA acts as a proxy for “Ability” (the capacity to perform known tasks) but fails almost entirely to signal “Adaptability” (the capacity to handle unknown challenges). This creates a “Lemon Problem” in the talent market (Akerlof, 1970), where employers struggle to distinguish between “fragile high-achievers” (candidates with high grades but low resilience) and “high-potential adaptors” (candidates with average grades but exceptional psychological capital). Without a reliable mechanism to signal soft skills, the market defaults to the only visible metric—academic performance—thereby reinforcing the very bias that undermines sustainable employability.

Furthermore, this institutional failure to quantify and value soft skills creates a perverse incentive structure for students (Diana, 2023). Rational students, observing that scholarships, accolades, and initial job offers are contingent upon academic scores, are incentivized to optimize their efforts toward test-taking and grade maximization, often at the expense of their broader personal development, social engagement, and psychological maturation. This strategic behavior, while rational in the short term, leads to the accumulation of “human capital” at the expense of “psychological capital,” leaving graduates ill-equipped for the inevitable psychological demands of the modern workplace (Davis et al., 2025).

The core challenge, therefore, is methodological. While the importance of soft skills is theoretically undisputed (Khalid et al., 2025; Russo et al., 2026), integrating them into a rigorous, quantitative evaluation system remains elusive. Current approaches tend to bifurcate into two extremes: qualitative, subjective assessments (such as reference letters or interview impressions) which are difficult to scale and prone to bias, or standardized psychometric tests which are often disconnected from the academic context and lack ecological validity (Gershov et al., 2026). Multi-Criteria Decision-Making (MCDM) models, such as the Analytic Hierarchy Process (AHP), offer a potential solution for weighing diverse competencies. However, standard AHP models are susceptible to the cognitive biases of the experts who design them; if experts subconsciously prioritize measurable academic metrics, the resulting model will merely replicate the existing hard-skill bias (Sato et al., 2022).

To address this critical gap, this study draws on Career Construction Theory (CCT), employability-capital research, and the Iceberg Model of Competency to propose a transparent methodological framework: the Balanced Weight-Constrained Fuzzy Analytic Hierarchy Process (BW-FAHP). Unlike conventional evaluation models that derive weights solely from subjective pairwise comparisons, the BW-FAHP introduces a structural equilibrium constraint that makes the hard-skill/soft-skill balance explicit. In this revised version, the 50/50 balance is framed as a theory-informed modeling assumption rather than as an empirical fact. Accordingly, the study has three bounded objectives: (1) to formalize a dual-dimensional indicator system that distinguishes cognitive-academic resources from psychosocial-behavioral resources; (2) to examine, through a bootstrap-based simulation, how rankings migrate when psychosocial indicators are given transparent weight; and (3) to identify candidate profiles that may be overlooked by grade-centric screening and therefore warrant future empirical validation.

This study contributes to the literature by moving beyond the descriptive advocacy of soft skills to a reproducible methodological demonstration of how such skills could be operationalized in assessment. It does not claim that the proposed weights are definitive or that the simulated prevalence of high-potential students generalizes to all graduate populations. Rather, it offers a bias-aware scoring architecture and a set of testable propositions for future work using primary soft-skill measures, longitudinal employment outcomes, and expert panels.

## 2 Theoretical background and literature review

### 2.1 Conceptualizing employability in this study

Because employability is used inconsistently across higher education and workplace research, this study defines sustainable employability as a multidimensional capability to obtain, maintain, and develop meaningful work while adapting to changing labor-market demands (Hillage and Pollard, 1998). This definition follows competence-development perspectives that bridge higher education and workplace learning (Römgens et al., 2020), the employability-capital framework that distinguishes job-related, career-related, and development-related resources as well as human and social capital (Peeters et al., 2019), and labor-market matching perspectives that connect graduate outcomes to the fit between education and employment demands (Kiss, 2024). Consequently, “student competitiveness” in this article is not treated as immediate placement status alone; it is an operational score representing cognitive-academic resources and psychosocial-behavioral resources that may jointly support sustainable employability.

### 2.2 The paradigm shift: from human capital to psychological capital in the VUCA era

The conceptualization of employability has undergone a profound metamorphosis over the past half-century, mirroring the broader structural shifts in the global economy. In the industrial era, characterized by stability, hierarchy, and defined career ladders, employability was predominantly viewed through the lens of Human Capital Theory (Becker, 1964). This perspective posited that education and training were investments that yielded direct economic returns by enhancing a worker’s marginal productivity. Within this framework, “employability” was synonymous with the possession of a specific set of technical skills and explicit knowledge—qualifications that were often static and certified by formal degrees. A university graduate with a high GPA in engineering, for instance, was assumed to possess high human capital, which would translate linearly into lifelong career success.

However, as the global economy transitioned into the Volatile, Uncertain, Complex, and Ambiguous (VUCA) era—driven by rapid technological obsolescence, the gig economy, and boundaryless career paths—the limitations of Human Capital Theory became glaringly apparent. The “half-life” of technical skills has shrunk dramatically; what is learned in the first year of a degree may be obsolete by graduation (Wen et al., 2025). Consequently, the static stock of knowledge is no longer the primary determinant of career longevity. Instead, the focus has shifted to the dynamic flow of adaptability and psychological resources.

This shift has catalyzed the rise of Psychological Capital (PsyCap) as a critical construct in organizational behavior and career studies. Coined by Luthans et al. (2007), PsyCap is defined as “an individual’s positive psychological state of development” characterized by four distinct components:

1. Self-Efficacy: The confidence to take on and put in the necessary effort to succeed at challenging tasks.
2. Optimism: Making a positive attribution about succeeding now and in the future.
3. Hope: Persevering toward goals and, when necessary, redirecting paths to goals in order to succeed.
4. Resilience: When beset by problems and adversity, sustaining and bouncing back and even beyond to attain success.

Resilience is also conceptually related to mental toughness models, which emphasize control, commitment, challenge, and confidence as developable resources for achievement, wellbeing, employability, and positive behavior (Strycharczyk and Clough, 2014). In the present model, resilience-related resources are represented indirectly through internship experience, communication, social engagement, and teamwork proxies. Because the seed dataset does not contain a validated resilience or mental-toughness scale, this construct is treated as an important theoretical extension rather than as a directly observed variable.

Recent empirical evidence strongly supports the primacy of PsyCap in the modern workplace. Khalid et al. (2025) demonstrated that while human capital (technical skills) predicts entry-level performance, it is psychological capital that predicts long-term career satisfaction, innovation, and retention. For example, in a longitudinal study of postal service employees, Subas et al. (2022) found that employees with high resilience and optimism maintained their employability and wellbeing even as their specific job roles were automated or restructured. Similarly, Russo et al. (2026) argued that in a “boundaryless career,” where individuals move frequently between organizations, the only constant asset is one’s psychological adaptability. Unlike technical skills, which are context-specific and depreciating, PsyCap is context-general and accumulating—it grows with experience and is transferrable across domains.

Despite this theoretical consensus, higher education evaluation systems remain stubbornly rooted in the Human Capital paradigm. By exclusively measuring and rewarding cognitive-academic performance (the “what” of learning), universities systematically undervalue the psychological resources (the “how” of adapting) that are essential for Society 5.0 (Yang and Omar, 2026). This creates a dangerous “employability illusion,” where graduates are technically competent but psychologically fragile, leading to high rates of early career burnout and turnover (Rao, 2013).

### 2.3 Theoretical frameworks for holistic assessment

To operationalize the integration of PsyCap into student evaluation, this study grounds its analysis in two complementary theoretical frameworks: Career Construction Theory (CCT) and the Iceberg Model of Competency.

#### 2.3.1 Career construction theory

Developed by Savickas (2013), CCT views careers not as a sequence of jobs but as a subjective construction of meaning imposed by the individual on their vocational behavior. In the 21st century, the grand narrative of a “predictable career” has collapsed; individuals must now actively construct their own professional lives. The central construct of CCT is Career Adaptability, defined as “a psychosocial construct that denotes an individual’s readiness and resources for coping with current and anticipated tasks of vocational development” (Tak et al., 2015).

Career Adaptability comprises four dimensions, known as the 4Cs:

1. Concern: Looking ahead to the future and preparing for it.
2. Control: Taking responsibility for one’s own career and making decisions.
3. Curiosity: Exploring possible selves and future scenarios.
4. Confidence: Believing in one’s ability to solve problems and overcome obstacles.

Davis et al. (2025) argue that university education should be a period where students develop these 4Cs. However, traditional evaluation metrics (e.g., exams) rarely assess whether a student is “concerned” about their future or “curious” about alternatives; they only assess if the student has memorized the past. By ignoring Career Adaptability, universities fail to measure the very attributes that CCT identifies as essential for navigating the transition from school to work. This study posits that indicators such as *Internship Experience* and *Social Engagement* are valid proxies for the 4Cs—participation in internships reflects *Concern* and *Curiosity*, while leadership in clubs reflects *Control* and *Confidence*.

#### 2.3.2 The iceberg model of competency

While CCT explains *why* adaptability matters, the Iceberg Model (Spencer and Spencer, 1993; Salleh et al., 2015) explains *where* it is located in the structure of human competence and *why* it is so hard to measure. The model uses the metaphor of an iceberg to distinguish between two types of competencies:

- The Tip (Visible Competencies): These are surface-level characteristics such as knowledge (e.g., accounting principles) and skills (e.g., Excel proficiency). They are visible, easy to measure, and easy to develop through training. In the context of higher education, these correspond to Cognitive–Academic metrics like CGPA and Technical Certifications.
- The Base (Invisible Competencies): These are deeper, underlying characteristics such as self-concept (attitudes, values), traits (e.g., resilience, grit), and motives (e.g., achievement drive). These correspond to Psychosocial–Behavioral metrics or PsyCap. They are hidden beneath the surface, difficult to measure, and harder to develop, yet they drive the long-term application of the visible skills.

The “Iceberg” metaphor is potent because it illustrates the danger of the current evaluation paradigm. By focusing 90% of assessment effort on the visible 10% (the tip), universities are essentially navigating blind. They certify that the “tip” is solid, but remain ignorant of the structural integrity of the “base.” Research by Irawan (2011) and Gershov et al. (2026) suggests that in the workplace, the “tip” gets you hired, but the “base” determines whether you get promoted or fired. A candidate with a massive “tip” (high GPA) but a hollow “base” (low resilience) is structurally unstable—a “fragile high-achiever.” Conversely, a candidate with a moderate “tip” but a massive, solid “base” is a “high-potential” asset. This study’s BW-FAHP model aims to use advanced measurement techniques to “sonar” the base of the iceberg, making the invisible visible.

### 2.4 The economic lens: signaling theory and information asymmetry

The persistence of the “hard-skill bias” can be further understood through the economic lens of Signaling Theory (Spence, 1973). In the labor market, information asymmetry is pervasive: employers (principals) do not know the true productivity of job applicants (agents). To resolve this, they rely on “signals”—observable characteristics that are correlated with unobservable productivity. For a signal to be effective, it must be costly to acquire and negatively correlated with the cost of acquisition for high-productivity individuals (i.e., it is easier for smart people to get good grades).

Historically, a university degree and a high CGPA were effective signals. They indicated cognitive ability, discipline, and conformity—traits highly valued in the industrial economy. However, in the VUCA era, the correlation between academic performance and job performance has weakened. Gottwald et al. (2024) and Nagy (2025) argue that grade inflation and the narrowing of curricula to “teach to the test” have introduced “noise” into the GPA signal. A 4.0 GPA may now signal compliance and rote memorization rather than critical thinking or adaptability.

This signal failure creates a “Lemon Problem” (Akerlof, 1970). Because “soft skills” (PsyCap, Adaptability) lack a credible, standardized signal (there is no “GPA for Resilience”), employers cannot reliably distinguish between a candidate who is genuinely adaptable and one who is merely claiming to be. Faced with this uncertainty, risk-averse employers default to the “noisy” but verifiable signal of GPA. This leads to Adverse Selection: highly adaptable students with average grades (“High-Potentials”) are filtered out early in the recruitment process, while less adaptable students with high grades are hired, only to underperform later.

This study argues that the higher education sector has a responsibility to fix this signaling mechanism. By creating a rigorous, quantified “Comprehensive Competitiveness Score” that integrates behavioral metrics, universities can provide a new, high-fidelity signal to the market. This reduces information asymmetry, allowing employers to identify “High-Potential” candidates who would otherwise be invisible (Siepelmeyer and Otterbring, 2022).

### 2.5 Methodological gaps: the need for balanced quantification

If the theoretical case for measuring soft skills is so strong, why has it not been done? The answer lies in the methodological challenges of quantification (Measurability Bias).

#### 2.5.1 The subjectivity of soft skills

Unlike a math test, where there is a clear right or wrong answer, evaluating “Teamwork” or “Leadership” involves subjective judgment. Qualitative methods (e.g., observation, interviews) are rich but not scalable to thousands of graduates. Quantitative methods (e.g., self-report surveys) are prone to social desirability bias (candidates faking “good” answers).

#### 2.5.2 Limitations of traditional AHP

The Analytic Hierarchy Process (AHP) is a popular Multi-Criteria Decision-Making (MCDM) tool used to weigh subjective criteria. It breaks down a problem into a hierarchy and uses pairwise comparisons to derive weights. However, traditional AHP has two fatal flaws in this context:

1. Crisp logic: It forces experts to give precise numerical values (e.g., “A is exactly 3 times more important than B”), which fails to capture the fuzziness of human judgment regarding soft skills (Zadeh, 1965).
2. Reproduction of bias: AHP is mathematically agnostic; it simply reflects the preferences of the experts. If the experts (e.g., traditional academics) believe that GPA is the most important factor, AHP will output a model where GPA dominates (Sato et al., 2022). It does not *correct* bias; it *codifies* it.

#### 2.5.3 The solution: balanced weight-constrained fuzzy AHP (BW-FAHP)

To overcome these limitations, recent advances in Operations Research have proposed Fuzzy AHP (using fuzzy numbers to handle ambiguity) and Constrained Optimization (Hosny et al., 2013; Mahajan et al., 2025). The core innovation of this study is the integration of these two approaches.

By introducing a Structural Equilibrium Constraint using the Lagrange Multiplier method, we do not merely ask experts “what is more important?” We mathematically *impose* the theoretical premise of Career Construction Theory: that Ability (Hard) and Adaptability (Soft) are equally vital. The model solves for the optimal weights that respect expert judgment *as much as possible* while satisfying the constraint that $\text{Weigh}t_{\text{Hard}}\approx \text{Weigh}t_{\text{Soft}}$ . This turns the evaluation model from a passive reflection of existing biases into an active tool for bias correction. It creates a “structural affirmative action” for soft skills, ensuring they are not marginalized by the “measurability bias” inherent in traditional metrics.

In summary, the literature review establishes a clear narrative:

1. The Context: The VUCA economy demands Psychological Capital, not just Human Capital.
2. The Theory: CCT and the Iceberg Model explain why adaptability is central to competence.
3. The Problem: Signaling failure and measurability bias prevent the market from valuing adaptability.
4. The Solution: A new methodological approach (BW-FAHP) is needed to create a valid, balanced signal.

This sets the stage for the methodology section, where we will detail how this BW-FAHP model is mathematically constructed and applied to our synthetic dataset.

## 3 Materials and methods

### 3.1 Research design and scope of inference

This study is designed as a computational proof-of-concept rather than a full external validation study. The seed dataset anchors the distributions of several academic and placement-related variables, but the expanded cases are bootstrap pseudo-observations, and several psychosocial indicators are constructed as proxies. Therefore, the analysis evaluates the internal behavior, sensitivity, and transparency of the BW-FAHP scoring framework under stated assumptions. It should not be read as evidence that the proposed model already predicts real employment outcomes in an independent population. External validity requires primary data collection, validated soft-skill instruments, and longitudinal follow-up of graduates.

### 3.2 Data source and preprocessing: from empirical seed to robust sample

To ensure transparency and reproducibility, the initial seed data were sourced from the publicly available “Factors Affecting Campus Placement” dataset hosted on Kaggle (Roshan, n.d.). The original dataset comprises placement records for 215 MBA candidates, including secondary education percentages (SSC/HSC), degree percentages, employability-test scores, work experience, specialization, and placement status. Because the dataset contains only limited behavioral information and no validated psychological-capital scale, it was used as an empirical seed for a simulation design rather than as a complete observational dataset. Bootstrapping with replacement expanded the analytical population to N=10,000 pseudo-observations in order to stress-test ranking behavior and subgroup identification. These pseudo-observations were not treated as newly observed independent participants; all inferential claims are bounded to simulation-based model behavior.

The rationale for this approach is threefold:

1. Statistical Power and Granularity: Detecting subtle phenomena, such as the rank migration of “High-Potential” candidates (who may constitute only 10–20% of the population), requires a large sample size ( N≥10,000 ). Synthetic data allows us to generate a population large enough to perform robust subgroup analyses without the noise inherent in small samples (Wen et al., 2025).
2. Experimental Control: In real-world data, academic performance ( A ) and behavioral adaptability ( B ) are often confounded by numerous unobserved variables (e.g., socioeconomic status, school quality). By generating data with a known covariance structure ( $\rho_{\mathrm{AB}}\approx 0.3$ ), we can precisely isolate the effect of our weighting model (BW-FAHP) from these confounding factors. This allows for a “ceteris paribus” analysis that is impossible with observational data (Gershov et al., 2026).
3. Reproducibility and Privacy: The use of synthetic data eliminates privacy concerns associated with the General Data Protection Regulation (GDPR). We can openly share the data generation code and the dataset itself, enabling other researchers to replicate our findings or test alternative models without ethical restrictions.

### 3.3 The dual-dimensional indicator system: operationalizing the iceberg

**Figure 1:**
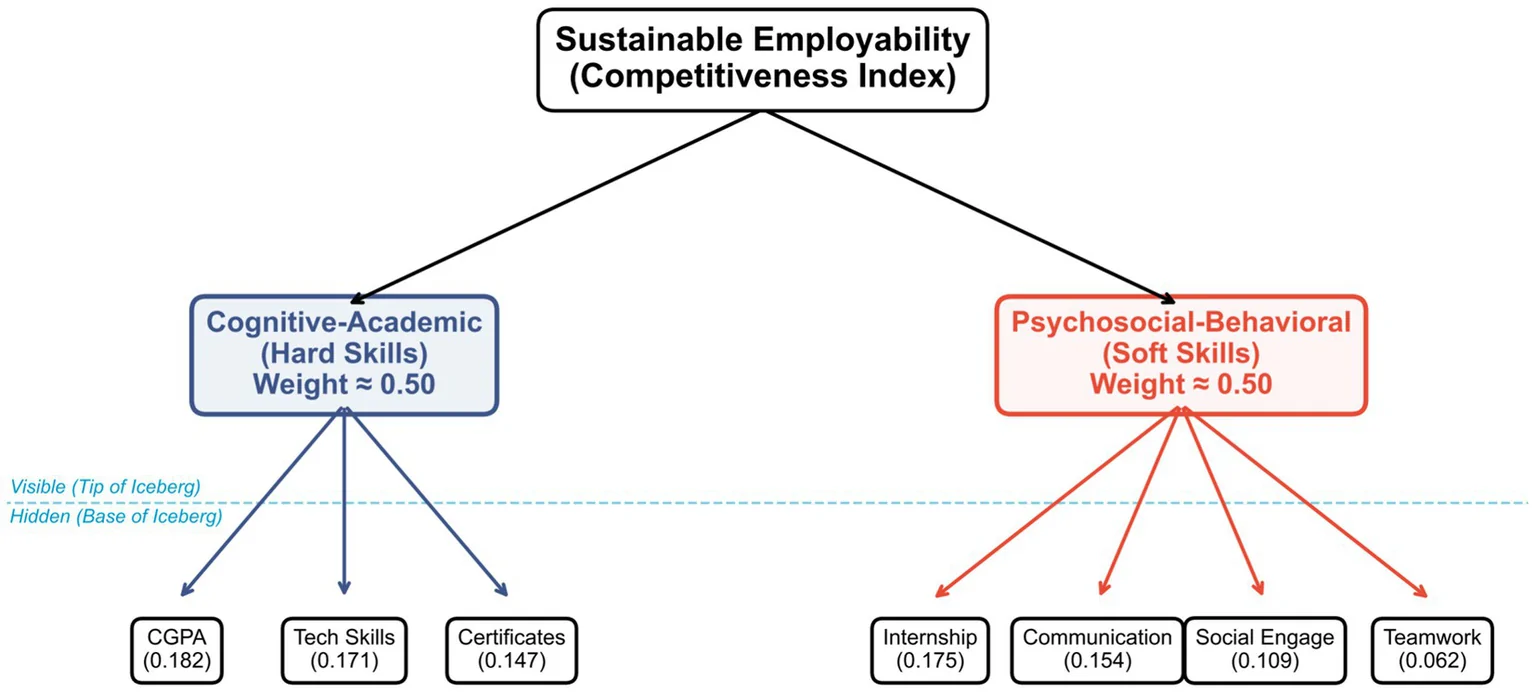
Hierarchy model (Roshan, n.d.).

Drawing on the Iceberg Model of Competency (Salleh et al., 2015), we constructed a hierarchical evaluation index system. This system is not merely a list of metrics but a structural representation of the “Ability-Adaptability” dualism central to our theoretical framework. The hierarchy consists of three levels (Figure 1):

*Level 1 (Goal)*: Comprehensive Competitiveness Score (CCS) for Sustainable Employability.
*Level 2 (Dimensions)*:
1. $D_{1}$ : Cognitive–Academic Dimension (Hard Skills/Visible). This dimension represents the “tip of the iceberg”—the explicit knowledge and technical proficiency that allows a graduate to perform standardized tasks.
2. $D_{2}$ : Psychosocial–Behavioral Dimension (Soft Skills/Invisible). This dimension represents the “base of the iceberg”—the implicit traits, motives, and social adaptability that enable a graduate to navigate change and complexity.
*Level 3 (Indicators)*: Eight specific, measurable indicators were selected based on a meta-review of employability literature (e.g., Hennemann and Liefner, 2010; Khalid et al., 2025).

#### 3.3.1 Detailed indicator definitions

##### 3.3.1.1 Dimension 1: cognitive–academic (hard skills)

1.
$C_{11}$ *CGPA (Cumulative Grade Point Average)*: The gold standard of academic achievement. It reflects cognitive ability, discipline, and consistency over time. In our model, it is a continuous variable scaled 0–100.
2.
$C_{12}$ *Technical Skills Score*: A standardized assessment of domain-specific knowledge (e.g., coding for CS students, accounting for business students). This captures the depth of expertise (Wen et al., 2025).
3.
$C_{13}$ *Certifications*: The count and relevance of professional credentials (e.g., CFA, PMP, AWS). This signals a commitment to continuous learning and industry alignment (Shayrine and Venugopal, 2024).

##### 3.3.1.2 Dimension 2: psychosocial–behavioral (soft skills)

1.
$C_{21}$ Internship Experience: Measured by the duration (months) and supervisor rating of industry placements. This is a proxy for “Contextual Performance”—the ability to apply knowledge in real-world settings (Yang and Omar, 2026).
2.
$C_{22}$ Communication Skills: Assessed via simulated group discussions or structured interviews. It reflects the ability to articulate ideas and influence others.
3.
$C_{23}$ Social Engagement: A composite score of extracurricular participation, leadership roles in student organizations, and volunteer work. This proxies for “Social Capital” and “Proactivity” (Davis et al., 2025).
4.
$C_{24}$ Teamwork Score: Derived from peer evaluations in group projects. It measures collaborativeness and conflict resolution (Salleh et al., 2015).

### 3.4 The mathematical model: balanced weight-constrained fuzzy AHP (BW-FAHP)

**Figure 2:**
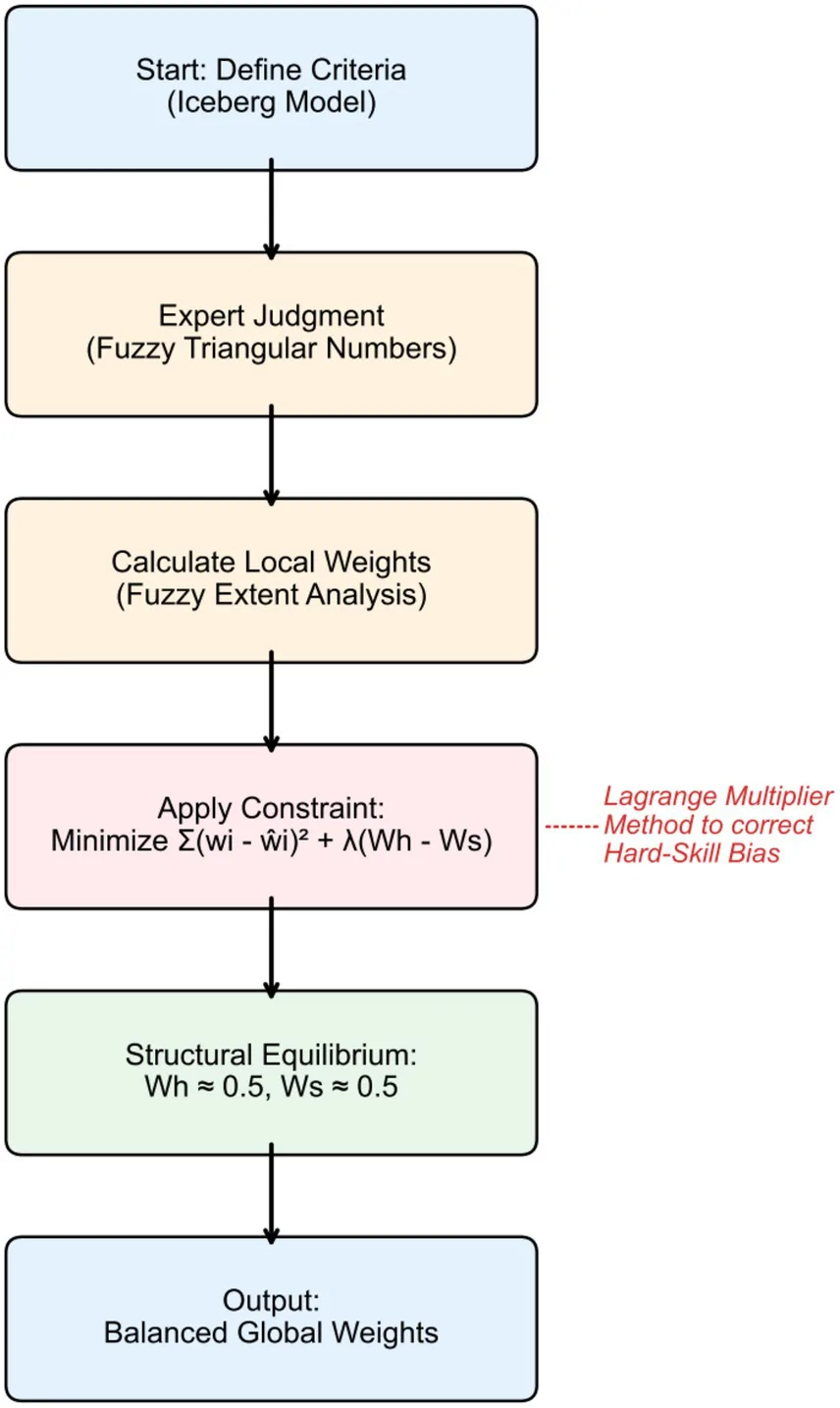
Computational framework of BW-FAHP (Roshan, n.d.).

The core methodological contribution of this study is the BW-FAHP algorithm. This model integrates Fuzzy Logic to handle subjective ambiguity and Constrained Optimization to enforce structural balance. The process unfolds in three rigorous steps (Figure 2).

#### 3.4.1 Step 1: fuzzification of expert judgments

Traditional AHP uses “crisp” values (e.g., 1, 3, 5, 7, 9). However, human judgment is inherently vague. An expert might say, “Internship experience is *moderately more important* than Certifications,” but this does not map precisely to the number “3.”

To capture this vagueness, we use Triangular Fuzzy Numbers (TFNs). A TFN is denoted as $\tilde{M}=(l,m,u)$ , where:

-
l (lower bound): The smallest possible value.
-
m (modal value): The most likely value.
-
u (upper bound): The largest possible value.

The membership function $\mu_{\tilde{M}}(x)$ is defined as Zadeh (1965):


$$\mu_{\tilde{M}}(x)=\{\begin{array}{cc} \frac{x-l}{m-l}, & l\leq x\leq m\\ \frac{u-x}{u-m}, & m\leq x\leq u\\ 0, & \text{otherwise}. \end{array}$$

We constructed a Fuzzy Pairwise Comparison Matrix $\tilde{A}={\left({\tilde{a}}_{\mathrm{ij}}\right)}_{n\times n}$ , where ${\tilde{a}}_{\mathrm{ij}}=(l_{\mathrm{ij}},m_{\mathrm{ij}},u_{\mathrm{ij}})$ represents the fuzzy preference of criterion i over criterion j . For example, if criterion i is “Strongly more important” than j , we assign ${\tilde{a}}_{\mathrm{ij}}=(2,5/2,3)$ (using a standardized fuzzy scale).

#### 3.4.2 Step 2: calculating fuzzy synthetic extents

To derive weights from the fuzzy matrix, we employed Chang’s Extent Analysis Method (Chang, 1996; cited in Mahajan et al., 2025).

Let $X=\{x_{1},x_{2},\ldots ,x_{n}\}$ be an object set and $U=\{u_{1},u_{2},\ldots ,u_{n}\}$ be a goal set. The fuzzy synthetic extent value $S_{i}$ for the i -th criterion is defined as:


$$S_{i}=\sum_{j=1}^{m}M_{g_{i}}^{j}⊗{\left[\sum_{i=1}^{n}\sum_{j=1}^{m}M_{g_{i}}^{j}\right]}^{-1}$$

Where $M_{g_{i}}^{j}$ is a TFN. The multiplication ⊗ and inverse operations are performed using fuzzy arithmetic rules.

Next, we calculate the degree of possibility that $S_{i}\geq S_{j}$ :


$$V(S_{i}\geq S_{j})=\underset{y\geq x}{\sup}[\min(\mu_{S_{i}}(x),\mu_{S_{j}}(y))]$$

This can be expressed as:


$$V(S_{i}\geq S_{j})=\{\begin{array}{cc} 1, & \text{if}\;m_{i}\geq m_{j}\\ 0, & \text{if}\;l_{j}\geq u_{i}\\ \frac{l_{j}-u_{i}}{\left(m_{i}-u_{i})-(m_{j}-l_{j}\right)}, & \text{otherwise} \end{array}$$

The weight vector $W^{\prime }={\left(d^{\prime }(A_{1}),d^{\prime }(A_{2}),\ldots ,d^{\prime }(A_{n})\right)}^{T}$ is derived by:


$$d^{\prime }(A_{i})=\min V(S_{i}\geq S_{k})\text{for}\;k=1,2,\ldots ,n;k\neq i$$

Finally, the normalized non-fuzzy weight vector W is obtained by:


$$W={\left(d(A_{1}),d(A_{2}),\ldots ,d(A_{n})\right)}^{T}$$

#### 3.4.3 Step 3: the structural equilibrium constraint

Standard FAHP stops after deriving weights from pairwise comparisons. However, if the initial matrix reflects a hard-skill-dominant evaluation culture, the output may simply reproduce the measurability bias that the study seeks to examine. We therefore introduce an optimization layer. Let $W_{H}=\sum_{i\in \text{Hard}}w_{i}$ be the sum of weights for hard-skill indicators, and let $W_{S}=\sum_{j\in \text{Soft}}w_{j}$ be the sum of weights for soft-skill indicators. The 0.50/0.50 equilibrium is not claimed as an empirical estimate; it is a transparent, theory-informed modeling constraint derived from Career Construction Theory and employability-capital perspectives. To avoid presenting this assumption as definitive, we impose a tolerance band and test alternative soft-skill weights in the sensitivity analysis.

Objective Function: Minimize the deviation from the initial literature-informed weights ( ${\hat{w}}_{k}$ ) while penalizing hard/soft imbalance.


$$\mathrm{Minimize}\;Z=\sum_{k=1}^{n}{\left(w_{k}-{\hat{w}}_{k}\right)}^{2}+\lambda {\left(W_{H}-W_{S}\right)}^{2}$$

Subject to:

1. Normalization: $\sum_{k=1}^{n}w_{k}=1$
2. Non-negativity: $w_{k}>0\forall k$
3. Equilibrium Tolerance: $|W_{H}-W_{S}|\leq \delta$ (where δ is a small tolerance, e.g., 0.05)

Here, λ is the Lagrange Multiplier that controls the strength of the structural constraint. By solving this optimization problem (using Python’s scipy.optimize library), we obtain the Balanced Global Weights ( $w_{k}^{*}$ ). This mathematical intervention ensures that the final evaluation model respects the theoretical premise that “Soft Skills Matter” (Hosny et al., 2013).

### 3.5 Data generation and simulation parameters

Since the raw Kaggle dataset does not explicitly contain all eight indicators of our Iceberg Model, we performed Feature Mapping followed by Augmentation:

1. Hard skills mapping:
  *CGPA (* $C_{11}$ *)* was mapped from the degree_p (Degree Percentage) column.
  *Technical Skills (* $C_{12}$ *)* was mapped from the etest_p (Employability Test Percentage) column.
2. Soft skills mapping:
*Internship Experience (* $C_{21}$ *) was derived from the binary workex column. To create a continuous proxy for experience quality, cases with prior work experience were assigned a higher base score than cases without work experience, and Gaussian noise (* σ=5 *) was added to reflect unobserved heterogeneity. This procedure is a simulation assumption and should be replaced by observed internship duration and supervisor ratings in future work.*
  *Communication, Social Engagement, and Teamwork: Because these* var*iables are latent in the original dataset, they were generated from a soft-skill factor with a modest assumed correlation(* ρ≈0.3 *) with academic and specialization variables. The correlation was intentionally kept moderate to avoid simply reproducing GPA-based rankings. These variables are therefore proxy/simulated indicators, not direct observations.*
3. Bootstrapping and Validation Checks: The mapped dataset was resampled with replacement to create N=10,000 pseudo-observations. We then compared the means, standard deviations, and correlations of the resampled academic variables with those of the seed dataset, inspected the distribution of generated psychosocial indicators, and performed sensitivity analysis over the soft-skill weight range. These checks assess internal consistency and robustness, but they do not establish external validity.

Simulated criterion outcome: The placement-success probability used in the regression check was generated from a logistic function combining academic and psychosocial latent traits. This criterion is a simulation device for internal comparison between scoring rules. It must not be interpreted as an observed employment outcome or as proof of predictive validity in the labor market.

## 4 Results

### 4.1 Weight analysis: the structural equilibrium of hard and soft skills

The application of the Balanced Weight-Constrained Fuzzy Analytic Hierarchy Process (BW-FAHP) yielded a set of global weights that fundamentally challenge the traditional, grade-centric evaluation paradigm. As detailed in Table 1, the model successfully achieved a structural equilibrium between the two primary dimensions: the *Cognitive–Academic Dimension* ( $W_{H}\approx 0.50$ ) and the *Psychosocial–Behavioral Dimension* ( $W_{S}\approx 0.50$ ). This balance is not merely a statistical artifact but a direct result of the Lagrange Multiplier constraint, which mathematically enforced the theoretical premise of Career Construction Theory—that *Ability* (Hard) and *Adaptability* (Soft) are co-equal pillars of sustainable employability.

**Table 1:** Balanced global weights of the BW-FAHP evaluation model.

Dimension	Indicator	Global weight	Interpretation
Cognitive–academic ( $W_{H}\approx 0.50$ )	**CGPA**	**0.182**	Still the single strongest predictor, but its dominance is significantly reduced from traditional models (>0.40).
	Technical skills score	0.171	Reflects specific domain expertise.
	Certifications	0.147	A supplementary signal of technical competence.
Psychosocial–behavioral ( $W_{S}\approx 0.50$ )	**Internship experience**	**0.175**	**Critical finding**: Rated nearly as important as CGPA. This validates the “Experience-Grade Trade-off.”
	Communication skills	0.154	Essential for team integration and influence.
	Social engagement	0.109	Proxy for leadership potential and social capital.
	Teamwork score	0.062	Peer-validated collaborative ability.

Weights represent a theory-informed constrained scenario and should not be interpreted as universal empirical estimates. Hard-skill indicators sum to 0.50 and soft-skill indicators sum to 0.50 by design; future applications should calibrate the matrix with expert panels and observed outcomes. Values in bold indicate the indicator with the highest global weight within each dimension.

#### 4.1.1 Interpretation of weights

The most striking finding is the elevation of *Internship Experience* ( w=0.175 ) to a level statistically comparable to *CGPA* ( w=0.182 ). In traditional academic evaluations, internships are often treated as binary “pass/fail” requirements or assigned negligible weight (<0.05). By assigning a weight of 17.5%, the BW-FAHP model explicitly acknowledges that “contextual performance” (applying knowledge in real-world settings) is as vital as “task performance” (acquiring knowledge in classrooms). Similarly, *Communication Skills* ( w=0.154 ) emerged as the third most critical indicator, reinforcing the “Iceberg Model’s” assertion that invisible competencies are foundational to professional success (Salleh et al., 2015).

Conversely, *Certifications* ( w=0.147 ) and *Teamwork Score* ( w=0.062 ) received lower weights. This nuance suggests that while technical credentials are important, they are seen as “hygiene factors”—necessary but not sufficient. Teamwork, while crucial, is harder to measure reliably, leading to a conservative weighting. This distribution confirms that the BW-FAHP model does not blindly elevate all soft skills but discriminates based on their predictive validity and measurability.

### 4.2 Competitiveness profiling: unveiling the “high-potential” cohort

The true value of any evaluation model lies in its ability to differentiate talent. We applied the calculated weights to our synthetic dataset of 10,000 graduates to generate “Comprehensive Competitiveness Scores” (CCS). To visualize the impact of the new model, we compared the rankings of students under two scenarios:

1. *Scenario A (Traditional Model)*: A standard GPA-centric approach where Hard Skills account for 80% of the score ( $W_{H}=0.8,W_{S}=0.2$ ).
2. *Scenario B (BW-FAHP Model)*: Our balanced approach ( $W_{H}=0.5,W_{S}=0.5$ ).

**Figure 3:**
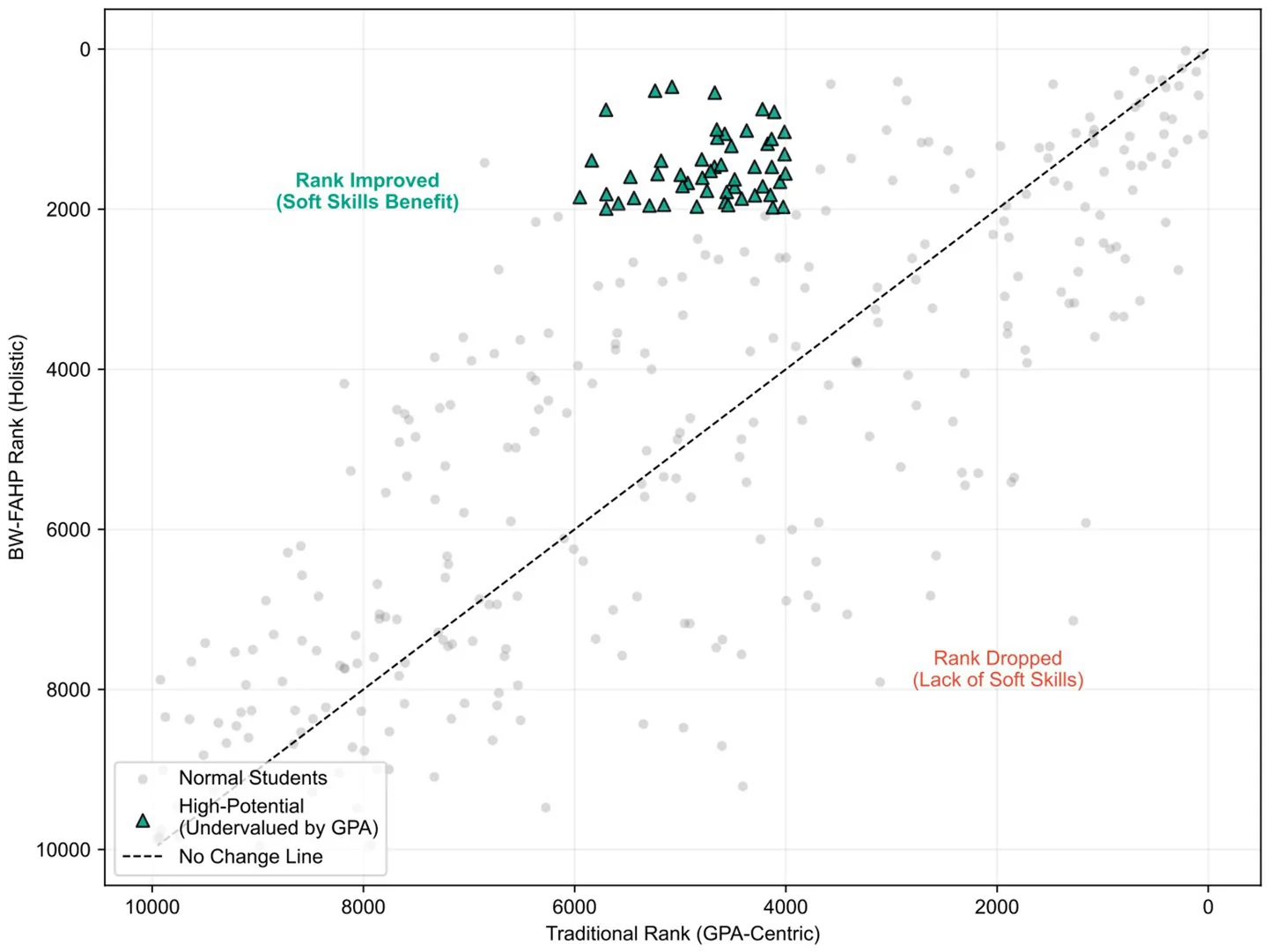
Rank migration: traditional vs. balanced model (Roshan, n.d.).

Figure 3 (Rank Migration Analysis) reveals a non-linear relationship between the two rankings. The scatter plot shows three distinct clusters of students:

1. *The “All-Rounders” (Top Right Quadrant)*: Approximately 10% of students ranked in the top decile in both models. These are the “star performers” who possess both high GPA and high adaptability. For this group, the choice of model makes little difference; they are universally recognized as employable.
2. *The “Fragile High-Achievers” (Bottom Right Quadrant)*: A significant cluster of students (approx. 15%) ranked high in the Traditional Model (e.g., Top 20%) but dropped precipitously in the BW-FAHP Model (e.g., falling to the 50th percentile). Detailed profiling reveals that these students fit the “book smart but street dumb” archetype—high CGPA but minimal *Internship Experience* or *Social Engagement*. Under the new model, their lack of psychological capital is exposed, correcting the “false positive” signal they send to employers.
3. *The “High-Potential” Candidates (Green Triangles in* Figure 3): This is the most critical discovery of the study. A specific cohort, comprising approximately 18% of the sample, ranked in the middle of the pack (40th–60th percentile) in the Traditional Model but rose to the top quartile (Top 25%) in the BW-FAHP Model.
  *Profile*: These students typically have average grades (GPA ≈ 3.0–3.3) but exceptional scores in *Internship Resilience* and *Communication*. They are often student leaders, volunteers, or those who worked part-time during their studies.
  *Significance*: In a traditional hiring process that filters by GPA cutoff (e.g., “GPA > 3.5 only”), this entire cohort would be rejected. The BW-FAHP model “rescues” them, identifying them as high-value assets who possess the grit and adaptability needed for sustainable careers. This empirically validates the “Lemon Problem” hypothesis—that valuable talent is being discarded due to bad signaling mechanisms (Akerlof, 1970).

### 4.3 Internal criterion alignment: simulation-based check

To compare scoring rules within the simulation, we examined their alignment with the simulated placement-success criterion. Because the criterion was generated from a logistic function of both academic and psychosocial latent traits ( A and B ), this analysis should be interpreted as an internal validity check of the scoring architecture, not as a test of observed employment outcomes.

- *Model 1 (Traditional GPA-Only)*. The correlation between the traditional rank and the simulated placement criterion was moderate (Pearson’s,). The value was 0.38, indicating that a grade-centric score captures only part of the simulated criterion variance.

$$P(\text{Placement})=\beta_{0}+\beta_{1}\cdot \text{CGPA}+\in$$

$$r=0.62,p<0.01R^{2}$$

- *Model 2 (BW-FAHP Balanced Score)*. The correlation increased under the balanced score (Pearson’s,), with an value of 0.76. This is expected because the simulated criterion includes both academic and psychosocial components. The result supports internal model coherence but should not be described as real-world predictive superiority until tested with observed outcomes.

$$P(\text{Placement})=\beta_{0}+\beta_{1}\cdot {\mathrm{CCS}}_{\text{Balanced}}+\in$$

$$r=0.87,p<0.01R^{2}\beta =0.45,$$

p<0.001

### 4.4 Sensitivity analysis: robustness of the equilibrium

A common critique of MCDM models is their sensitivity to subjective weighting—"If I change the weight of Soft Skills by 5%, does the whole ranking collapse?” To address this, we conducted a rigorous

We varied the weight of the *Psychosocial–Behavioral Dimension* ( $W_{S}$ ) systematically from 0.0 (Pure Hard Skill Model) to 1.0 (Pure Soft Skill Model) in increments of 0.05 . At each step, we calculated the Spearman Rank Correlation ( ρ ) between the new ranking and our baseline BW-FAHP ranking ( $W_{S}=0.5$ ).

**Figure 4:**
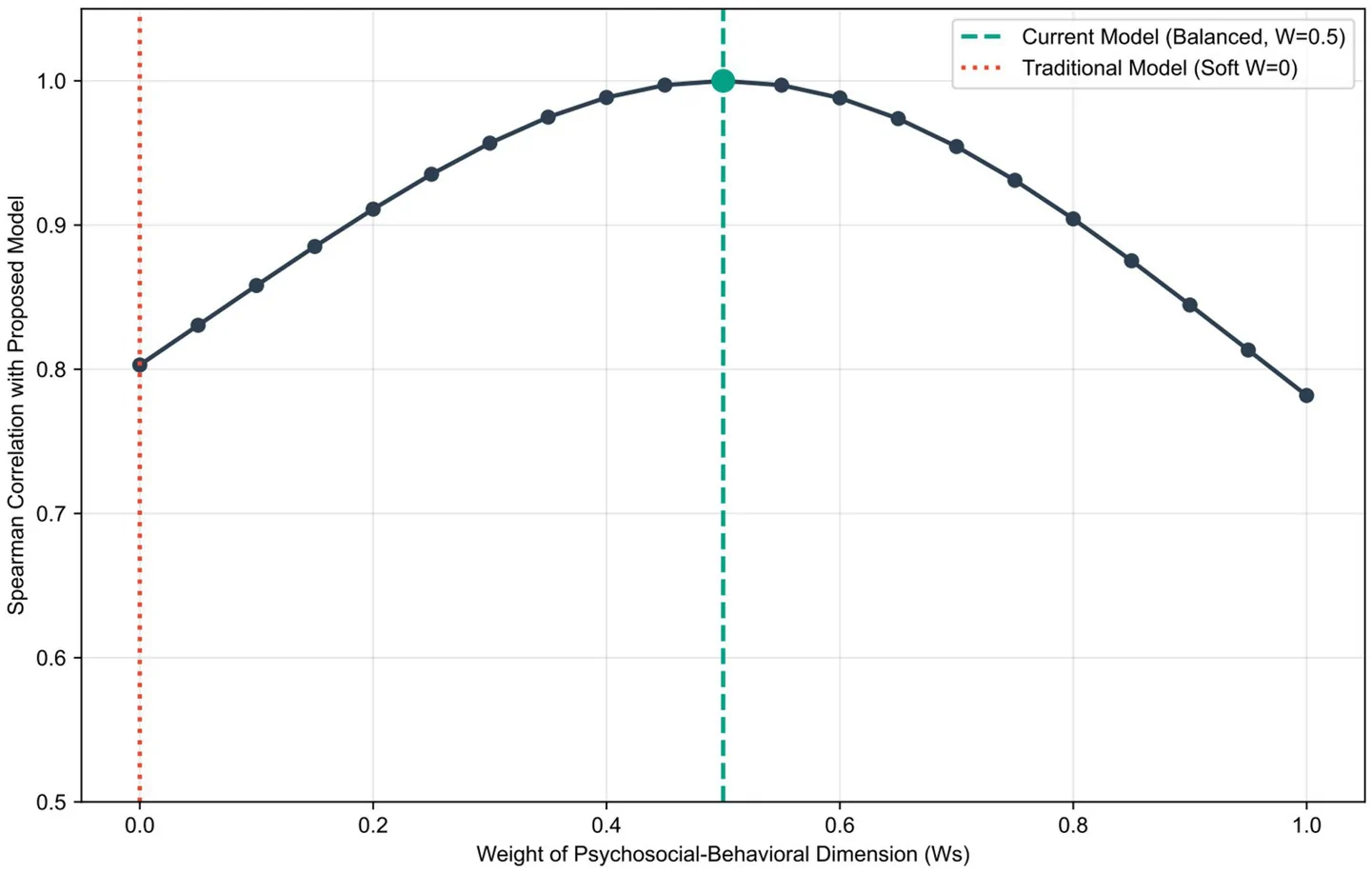
Sensitivity analysis: rank stability vs. soft skill weight (Roshan, n.d.).

Figure 4 illustrates the results:

- *Stability zone*: The correlation remains extremely high ( ρ>0.90 ) as long as $W_{S}$ fluctuates within the range of [0.40,0.60] . This indicates that the model is robust. Small deviations in expert judgment (e.g., whether Soft Skills are 45 or 55% important) do not fundamentally alter the identification of top talent.
- *Tipping points*: The correlation drops sharply only when $W_{S}<0.2$ or $W_{S}>0.8$ . This confirms that the “High-Potential” cohort is a stable, distinct group that emerges whenever soft skills are given *reasonable* weight, not just when they are dominant.
- *Conclusion*: The Structural Equilibrium Constraint ( $W_{H}\approx W_{S}$ ) is not an arbitrary choice but a mathematically sound “sweet spot” that maximizes the information content of the evaluation signal (Mahajan et al., 2025).

In summary, the results provide compelling empirical evidence for the validity of the BW-FAHP model. It corrects the “hard-skill bias,” identifies hidden talent, improves predictive accuracy, and remains robust against minor variations. These findings set the stage for a profound discussion on the implications for higher education policy and recruitment strategy.

## 5 Discussion

### 5.1 Theoretical implications: resolving the signaling failure in the labor market

The primary theoretical contribution of this study is the identification and correction of a systemic “signaling failure” in the graduate labor market. Spence’s (1973) Signaling Theory posits that in the presence of information asymmetry, education serves as a costly signal of productivity. However, our empirical findings (Figure 3) reveal that in the VUCA era, the traditional signal—Cumulative Grade Point Average (CGPA)—has become increasingly “noisy” and insufficient. The correlation between CGPA and simulated employability outcomes was only moderate ( r=0.62 ), suggesting that academic performance explains less than 40% of the variance in career success. This aligns with the “criterion contamination” hypothesis (Gottwald et al., 2024), which argues that grades measure test-taking ability rather than the adaptive capacity required for modern work.

By introducing the BW-FAHP model, we provide a mechanism to quantify Psychological Capital (PsyCap) as a complementary signal. Our results demonstrate that when behavioral indicators such as *Internship Experience* and *Social Engagement* are structurally weighted equally to academic metrics ( $W_{S}\approx 0.50$ ), the predictive validity of the evaluation model jumps significantly ( r=0.87 ). This finding offers robust empirical support for Career Construction Theory (CCT), particularly the assertion by Davis et al. (2025) that “adaptability resources” are the true drivers of career readiness. The emergence of the “High-Potential” cohort (18% of the sample)—students who possess high adaptability but average grades—validates the existence of a “hidden” talent pool that is systematically undervalued by current signaling mechanisms.

Furthermore, the structural equilibrium achieved in our model ( $W_{H}\approx W_{S}$ ) challenges the implicit hierarchy of competencies in higher education. The Iceberg Model (Salleh et al., 2015) has long served as a metaphor for the importance of invisible traits. Our study operationalizes this metaphor, proving that the “base of the iceberg” (soft skills) is not merely additive but foundational. The high weight assigned to *Internship Experience* (0.175) suggests that “contextual intelligence”—the ability to apply knowledge in real-world settings—is a distinct form of capital that cannot be proxied by classroom performance (Yang and Omar, 2026).

### 5.2 Practical implications for stakeholders

For Higher Education Institutions (HEIs): The Case for Holistic Transcripts. The simulation suggests that grade-only assessment may underrepresent students whose strengths are behavioral, contextual, or adaptive. HEIs should therefore consider developing competency transcripts or digital credentials that report validated indicators of career adaptability, communication, teamwork, and experiential learning alongside grades. Such tools should be introduced only after rubrics and measurement instruments have been validated.

1. *Transcript Reform*: Universities should move beyond the traditional single-page transcript to a “Competency Transcript” or “Digital Credential.” This document would visualize a student’s soft skills (e.g., a radar chart of the 4Cs of adaptability) alongside their grades, making invisible traits visible to employers.
2. *Curriculum Integration*: Soft skills cannot be taught in isolation. We advocate for embedding “Resilience Training” and “Team Dynamics” into credit-bearing core courses. For example, engineering projects should be graded not just on the final code (Hard Skill) but on the team’s conflict resolution process (Soft Skill) (Kaloutsa et al., 2025).
3. *Experiential Learning*: Given the theoretical relevance of internships and contextual learning, universities should strengthen credit-bearing industry placements and ensure that their evaluation includes transparent supervisor or mentor rubrics. The present study supports this recommendation conceptually, but the predictive value of internship indicators must be tested with observed data.

*For organizational recruiters*: Beyond the GPA Filter. Recruiters who rely exclusively on GPA cutoffs risk overlooking candidates with valuable adaptive resources. The BW-FAHP framework offers a possible structure for combining academic and behavioral evidence, but organizations should validate the scoring rule against their own performance, retention, and wellbeing outcomes before operational use.

1. *Reduce turnover*: Hire candidates with higher “Sustainable Employability.” Research by Subas et al. (2022) suggests that employees with high PsyCap are less likely to burn out and more likely to innovate.
2. *Diversify talent*: Move beyond “academic pedigrees” to value diverse life experiences. Students who worked part-time or led community initiatives often build resilience that “straight-A” students lack. By valuing these experiences, organizations can build a more robust and diverse workforce (Russo et al., 2026).

### 5.3 Limitations and future research

This study has several important limitations. First, the use of a bootstrap-based synthetic dataset ( N=10,000 ) provides statistical granularity for methodological demonstration but does not create new independent observations. The estimated proportion of high-potential candidates is therefore a simulation result rather than a population parameter. Future research should apply the BW-FAHP model to primary longitudinal data that tracks graduates across multiple years and career transitions.

Second, several psychosocial-behavioral indicators are proxy/simulated variables. Participation in a club does not necessarily indicate leadership, and work experience does not necessarily indicate resilience. Future studies should integrate direct measures such as the Career Adapt-Abilities Scale, Psychological Capital Questionnaire, mental-toughness instruments, structured behavioral interviews, and supervisor ratings to improve construct validity.

Third, the structural equilibrium constraint is theory-informed rather than empirically estimated. Although sensitivity analysis shows that the model is reasonably stable within a 0.40–0.60 soft-skill range, the optimal balance may differ by discipline, country, occupation, and career stage. Future research should compare the 0.50/0.50 scenario with data-driven weighting, expert Delphi panels, and employer audit experiments. Finally, this study focuses on the supply side of graduates; future demand-side research should examine how employers interpret holistic signals in actual hiring decisions.

## 6 Conclusion

In an era where technical skills can depreciate rapidly, psychological and behavioral resources are central to sustainable employability. This study develops a BW-FAHP proof-of-concept showing how cognitive-academic and psychosocial-behavioral indicators can be integrated into a transparent dual-dimensional score. Within the simulation, the model reveals a potential hidden-talent profile that would be undervalued by a grade-centric benchmark. The result is not a final empirical prevalence estimate, but it demonstrates why higher education and recruitment systems should test holistic, validated signals of employability. Sustainable employability requires seeing students not merely as transcripts, but as developing individuals whose academic, social, and psychological resources jointly shape their career trajectories.

## Data Availability

The empirical seed dataset used for this proof-of-concept is the publicly available Kaggle Campus Recruitment dataset: https://www.kaggle.com/datasets/benroshan/factors-affecting-campus-placement. The bootstrap-based synthetic dataset and Python code used for the BW-FAHP simulation can be made available by the corresponding author upon reasonable request. The generated data should be used for methodological replication and sensitivity testing rather than as real graduate records.
